# Probing the sensitivity of ab initio multiple spawning to its parameters

**DOI:** 10.1007/s00214-023-03004-w

**Published:** 2023-07-28

**Authors:** Yorick Lassmann, Basile F. E. Curchod

**Affiliations:** Centre for Computational Chemistry, School of Chemistry, Cantock’s Close, University of Bristol, Bristol, BS8 1TS UK

## Abstract

Full multiple spawning (FMS) offers a strategy to simulate the nonadiabatic dynamics of molecular systems by describing their nuclear wavefunctions by a linear combination of coupled trajectory basis functions (TBFs). Applying a series of controlled approximations to the full multiple spawning (FMS) equations leads to the *ab initio* multiple spawning (AIMS), which is compatible with an on-the-fly propagation of the TBFs and an accurate description of nonadiabatic processes. The AIMS strategy and its numerical implementations, however, rely on a series of user-defined parameters. Herein, we investigate the influence of these parameters on the electronic-state population of two molecular systems— trans-azomethane and a two-dimensional model of the butatriene cation. This work highlights the stability of AIMS with respect to most of its parameters, underlines the specific parameters that require particular attention from the user of the method, and offers prescriptions for an informed selection of their value.

**Supplementary Information:**

The online version contains supplementary material available at 10.1007/s00214-023-03004-w.

## Introduction

Finding a practical solution to the time-dependent Schrödinger equation for a molecular system constitutes a challenge that has met almost a century of continued effort. As the wave equation is analytically unsolvable for molecular systems containing more than two particles, approximations are the *sine qua non* of a practical use of the Schrödinger equation in chemistry. In this context, the developments proposed by Born and Oppenheimer [1] can be seen as stepping stones for theoretical and computational chemistry. Their approximation—separating the nuclear motion from the electronic one—brought forth the concept of potential energy surfaces (PESs) for molecules and is seminal in explaining most phenomena in (ground-state) chemistry. However, describing the dynamics of a molecule prepared in an excited electronic state as a result of light absorption, for example, leads to a breakdown of the Born–Oppenheimer approximation since nuclear and electronic motions become strongly coupled. Without the Born–Oppenheimer approximation, the complexity of the molecular dynamics increases dramatically because the coupled electron-nuclear, i.e., nonadiabatic dynamics requires to account for subtle nuclear quantum effects and coupling between multiple electronic states. The complexity thus required in simulating the photochemistry or photophysics of a molecule has provided fertile ground for the development of a plethora of methods for nonadiabatic molecular dynamics [2–4].

Methods for nonadiabatic molecular dynamics often differ by their representation of the (time-dependent) nuclear wavefunctions or densities: a nearly-exact representation using single-particle wavefunctions [5–7], a swarm of independent [8–10] or coupled [11–13] trajectories, or a basis set of traveling Gaussians, also known as trajectory basis functions (TBFs) [14–16]. The full multiple spawning (FMS) framework [17, 18] makes use of the last representation and expands the nuclear wavefunctions into a basis set of TBFs that move along classical paths on single adiabatic surfaces and whose amplitudes are fully coupled via the time-dependent Schrödinger equation. To describe the transfer of amplitude between nuclear wavefunctions on different electronic states, FMS allows the basis of TBFs to grow via the so-called spawning algorithm whenever two or more electronic states become coupled due to nuclear motion (see below for more details on the FMS method).

While FMS has been shown to reproduce exact quantum dynamics for model systems [19, 20], the method requires knowledge of the PESs and other electronic-structure quantities over the full nuclear configuration space for the propagation of the TBFs amplitudes. Consequently, FMS becomes prohibitively expensive for molecules in their full dimensionality. To address this curse of dimensionality, an *on-the-fly* variant of FMS, called *ab initio* multiple spawning (AIMS) [21], was derived based on two central approximations— the saddle-point approximation of order zero (SPA0) and the independent first-generation approximation (IFGA), both of which will be described in Sect. 2.1.3. AIMS has been successfully used to simulate the photodynamics of small- to medium-size molecules (see Ref. [16] for a list), or benchmark other methods for nonadiabatic dynamics like trajectory surface hopping (TSH) [22, 23].

As any AIMS practitioner may have realized, this technique depends on a wide range of parameters, some of which are directly related to the method itself, while others are connected to its numerical implementation. Some key parameters have been subjected to a detailed analysis previously—the widths of the TBFs [24] or the spawning thresholds [25]—but the influence of parameters on the resulting AIMS nonadiabatic dynamics has until now only been studied on simple model systems. In this work, we offer a comprehensive and pragmatic survey of the parameters involved in the AIMS method and their individual influence on the nonadiabatic dynamics of two molecular systems, a two-dimensional two-state model of the butatriene cation and trans-azomethane in its full dimensionality. We highlight the isolated effect of each parameter on the resulting AIMS dynamics, spotlight those parameters that need special attention and their converging behavior, and overall show the stability of the AIMS methods with respect to its parameters.

## Methods

### Brief overview of full and ab initio multiple spawning

We propose in the following a brief overview of the FMS and AIMS methods for nonadiabatic dynamics [17–19, 21]. For a more detailed account of these techniques, the interested reader is referred to Refs. [16, 26, 27].

#### Full multiple spawning

FMS aims to practically solve the time-dependent Schrödinger equation for a molecular system, containing $N_\textrm{el}$ electrons and $N_\textrm{n}$ nuclei,

1 $$\begin{aligned} i \frac{\partial }{\partial t}\Psi (\textbf{r}, \textbf{R}, t) = \hat{H}(\textbf{r}, \textbf{R})\Psi (\textbf{r}, \textbf{R},t) \, , \end{aligned}$$

where $\Psi (\textbf{r}, \textbf{R},t)$ is the total molecular wavefunction with coordinates of electrons labeled by $\textbf{r}=(\textbf{r}_1,\textbf{r}_2,\dots ,\textbf{r}_{N_\textrm{el}})$ and those of the nuclei by $\textbf{R}=(\textbf{R}_1,\textbf{R}_2,\dots , \textbf{R}_{N_\textrm{n}})$. Note that atomic units are used throughout this work. $\hat{H}(\textbf{r}, \textbf{R}) = \hat{T}_\textrm{n}(\textbf{R}) +\hat{\mathcal {H}}_\textrm{el}(\textbf{r}, \textbf{R})$ is the molecular Hamiltonian, composed of the kinetic energy operator for the nuclei and an electronic contribution, $\hat{\mathcal {H}}_\textrm{el}(\textbf{r}, \textbf{R})$, which groups the kinetic energy operator for the electrons and all the interaction potentials between electrons and nuclei. By solving the eigenvalue problem of this electronic contribution, the PESs emerge from the parametric dependency of the eigenvalues—commonly referred to as adiabatic electronic energies or $E_{J}^\textrm{el}(\textbf{R})$—on the nuclear configuration space. Furthermore, the eigenfunctions $\Phi _J(\textbf{r}; \textbf{R})$, the so-called electronic wavefunctions, can be used as a basis in which to expand the total molecular wavefunction, resulting in what is known as its Born–Huang representation,

2 $$\begin{aligned} \Psi (\textbf{r}, \textbf{R},t) = \sum _J^{\infty } \Phi _J(\textbf{r}; \textbf{R})\chi _J(\textbf{R},t) \,. \end{aligned}$$

$\chi _J(\textbf{R},t)$ plays the role of a time-dependent expansion coefficient, commonly associated with a time-dependent nuclear wavefunction labeled by the electronic state *J*.

FMS proposes to represent the time-dependent nuclear wavefunctions $\chi _J(\textbf{R},t)$ in terms of travelling multidimensional Gaussian functions (the TBFs)

3 $$\begin{aligned} \chi _J(\textbf{R}, t)=\sum _k^{N_\textrm{TBFs}^J(t)}C_k^{(J)}(t)\tilde{\chi }_k^{(J)}\left( \textbf{R};\overline{\textbf{R}}_k^{(J)}(t),\overline{\textbf{P}}_k^{(J)}(t), \varvec{\alpha }, \overline{\gamma }_k^{(J)}(t) \right) \, . \end{aligned}$$

That is, FMS expands each nuclear wavefunction $\chi _J(\textbf{R}, t)$ as a linear combination of $N_\textrm{TBFs}^J(t)$ multidimensional Gaussian functions $\tilde{\chi }_k^{(J)}\left( \textbf{R};\overline{\textbf{R}}_k^{(J)}(t),\overline{\textbf{P}}_k^{(J)}(t), \varvec{\alpha }, \overline{\gamma }_k^{(J)}(t) \right)$, each of them associated with a time-dependent complex coefficient $C_k^{(J)}(t)$ (*k* labels a specific TBF, evolving in electronic state *J*) and centred at $\overline{\textbf{R}}_k^{(J)}(t)$ and $\overline{\textbf{P}}_k^{(J)}(t)$ in position and momentum space, respectively. $\varvec{\alpha }$ symbolizes the width matrix and $\overline{\gamma }_k^{(J)}(t)$ a phase with semiclassical origins. It is important to stress that the TBFs are not fixed in phase space but will be moving over time along classical trajectories. We will return to the time dependence of $N_{TBFs}^J(t)$ when we discuss the so-called spawning algorithm.

Each multidimensional TBF is expressed as a product of one-dimensional Gaussian functions,

4 $$\begin{aligned}{} & {} \tilde{\chi }_k^{(J)}\left( \textbf{R};\overline{\textbf{R}}_k^{(J)}(t),\overline{\textbf{P}}_k^{(J)}(t), \varvec{\alpha }, \overline{\gamma }_k^{(J)}(t)\right) \nonumber \\{} & {} \quad =e^{i\overline{\gamma }_k^{(J)}(t)}\prod _{\rho }^{3N_\textrm{n}} \tilde{\chi }_{k\rho }^{(J)}\left( R_\rho ;\overline{R}_{k\rho }^{(J)}(t),\overline{P}_{k\rho }^{(J)}(t), \alpha _{\rho }\right) \,, \end{aligned}$$

where $\rho$ runs over all $3N_\textrm{n}$ nuclear coordinates, and each one-dimensional Gaussian function is defined by

5 $$\begin{aligned}{} & {} \tilde{\chi }_{k\rho }^{(J)}\left( R_\rho ;\overline{R}_{k\rho }^{(J)}(t),\overline{P}_{k\rho }^{(J)}(t), \alpha _{\rho }\right) \nonumber \\{} & {} \quad = \left( \frac{2\alpha _{\rho }}{\pi } \right) ^{1/4} \exp \left[ -\alpha _{\rho } \left( R_\rho - \overline{R}_{k\rho }^{(J)} \right) ^2 +i \overline{P}_{k\rho }^{(J)} \left( R_\rho - \overline{R}_{k\rho }^{(J)} \right) \right] \,. \end{aligned}$$

Replacing the nuclear wavefunctions in the Born–Huang molecular wavefunction (Eq. (2)) by their representation in terms of TBFs (Eq. (3)) leads to an FMS Ansatz for the molecular wavefunction. By inserting this Ansatz into the time-dependent Schrödinger equation (Eq. (1)) and doing some algebra (see Refs. [16, 26, 27] for details), we are left with a set of (coupled) equations of motion for the time-dependent coefficients

6 $$\begin{aligned} \frac{d \textbf{C}^I}{dt}=-i\left( \textbf{S}^{-1}\right) _{II}\left[ \left( \textbf{H}_{II} - i \dot{\textbf{S}}_{II}\right) \textbf{C}^I + \sum _{J\ne I}^\infty \textbf{H}_{IJ}\textbf{C}^J \right] \, . \end{aligned}$$

The overlap matrices have elements $\left( \textbf{S}\right) ^{II}_{k,k'}=\langle \tilde{\chi }_{k}^{(I)} | \tilde{\chi }_{k'}^{(I)} \rangle _{\textbf{R}}$ and $\left( \dot{\textbf{S}}\right) ^{II}_{kk'}=\langle \tilde{\chi }_{k}^{(I)} | \frac{\partial }{\partial t}\tilde{\chi }_{k'}^{(I)} \rangle _{\textbf{R}}$ and the Hamiltonian matrix element between TBF *k* evolving in state *I* and TBF $k'$ evolving in state *J* is given by

7 $$\begin{aligned} \left( \textbf{H}\right) ^{IJ}_{k ,k' }&= \langle \Phi _I \tilde{\chi }_{k }^{(I)} | \hat{H} | \tilde{\chi }_{k' }^{(J)} \Phi _J \rangle _{\textbf{r},\textbf{R}} \nonumber \\&= \langle \tilde{\chi }_{k }^{(I)} | \hat{T}_n | \tilde{\chi }_{k' }^{(J)} \rangle _{\textbf{R}} \delta _{IJ} + \langle \tilde{\chi }_{k }^{(I)} | E_J^{el} | \tilde{\chi }_{k' }^{(J)} \rangle _{\textbf{R}} \delta _{IJ} \nonumber \\&- \sum _\rho ^{3N_n} \frac{1}{2M_\rho } \langle \tilde{\chi }_{k }^{(I)} | \langle \Phi _I | \frac{\partial ^2}{\partial R^2_\rho }|\Phi _J \rangle _{\textbf{r}} | \tilde{\chi }_{k' }^{(J)} \rangle _{\textbf{R}} \nonumber \\&\quad - \sum _\rho ^{3N_n} \frac{1}{M_\rho } \langle \tilde{\chi }_{k }^{(I)} | \langle \Phi _I | \frac{\partial }{\partial R_\rho }|\Phi _J \rangle _{\textbf{r}} \frac{\partial }{\partial R_\rho } | \tilde{\chi }_{k' }^{(J)} \rangle _{\textbf{R}}\,. \end{aligned}$$

If the two TBFs considered are in the same electronic state, their coupling is determined by the first three terms on the r.h.s. of Eq. (7): a term containing the nuclear kinetic energy operator, another one the electronic energy, and finally a correction term deriving from the diagonal part ($I=J$) of the second-order nonadiabatic coupling. When the two TBFs evolve in different electronic states, they are coupled *via* the third term on the r.h.s of Eq. (7), i.e., the off-diagonal contribution of the second-order nonadiabatic coupling and the last term that depends on the nonadiabatic coupling vectors, $\textbf{d}_{IJ}(\textbf{R})=\langle \Phi _I | \frac{\partial }{\partial \textbf{R}}|\Phi _J \rangle _{\textbf{r}}$.

#### Spawning algorithm

A crucial ingredient of the FMS framework is the possible addition of new TBFs to accurately describe nonadiabatic processes. This process, called spawning, is the reason why the number of TBFs in Eq. (3), $N_\textrm{TBFs}^J(t)$, is time-dependent. The spawning process occurs in FMS in the following way. (i) The excited-state dynamics is initiated from a certain number of initial (ancestor) TBFs, whose nuclear positions and momenta are sampled to reproduce the initial nuclear wavefunction of the molecular system. (ii) Each TBF follows its own classical trajectory in a given electronic state and monitors, at each time step, the value of an indicator of nonadiabaticity with any other electronic states—this indicator can be the norm of nonadiabatic coupling vectors or their projection on the TBF nuclear velocity. (iii) When a TBF, which we refer to as the parent TBF, enters a region of configuration space where the value of the indicator is larger than a predefined threshold, the FMS dynamics enters the *spawning mode*. The spawning mode is intended to find the best location to create a new TBF on the coupled electronic state. (iv) The propagation of the complex amplitudes is suspended, but the classical dynamics of the parent TBF is continued until a maximum of the nonadiabaticity indicator is located. (v) A new TBF, the so-called child TBF, is created on the coupled state at the position of the maximum located in step (iv), with a complex coefficient set to zero. (vi) Both TBFs are backpropagated in time until the beginning of the spawning mode. It is worth noting that the new TBF will have a different dynamics from its parent as it is propagated in another electronic state. (vii) Once both TBFs have been propagated back to the initial time when the spawning mode was triggered, the FMS dynamics restarts with the inclusion of the new TBF in the equations of motion for the complex amplitudes, *i.e.,* the matrices in Eq. (6) are augmented by a new TBF. The parent TBF will have a maximum overlap with the child TBF in the region with a strong nonadiabatic coupling, ensuring a proper description of the transfer of nuclear amplitude between the two coupled electronic states. For more information about the spawning algorithm, the interested reader is referred to Refs. [16, 27, 28].

#### Ab initio multiple spawning

Let us now focus on the difference between the full and ab initio multiple spawning methods. The evaluation of the Hamiltonian matrix elements, as defined in Eq. (7), requires global knowledge of the electronic energies and nonadiabatic coupling terms over the full configuration space because of the integration involved, and hampers the use of FMS for molecular systems in their full dimensionality. To alleviate this problem, the AIMS method introduces two main approximations to the FMS equations of motion, aiming to simplify the evaluation of matrix elements. These two approximations make it possible to perform AIMS dynamics on-the-fly [21, 29, 30].

The first approximation consists in approximating the Hamiltonian matrix elements by performing a Taylor expansion of the electronic-structure quantities at the centroid position between the two considered TBFs (their average position) and retaining only the term of order zero. This approximation, coined the SPA0, implies that the electronic-structure quantity can be considered constant in the region of configuration space where the overlap between the two TBFs considered is non-zero. Hence, the Hamiltonian matrix element between TBF *k* evolving in state *I* and TBF $k'$ evolving in state *J* is approximated in AIMS by

8 $$\begin{aligned}&\left( \textbf{H}\right) ^{IJ}_{k ,k' } \approx \langle \tilde{\chi }_{k }^{(I)} | \hat{T}_n | \tilde{\chi }_{k' }^{(J)} \rangle _{\textbf{R}} \delta _{IJ} + E_J^{el}(\overline{\textbf{R}}_{k,k'}^{(IJ)}) \langle \tilde{\chi }_{k }^{(I)} | \tilde{\chi }_{k' }^{(J)} \rangle _{\textbf{R}} \delta _{IJ} \nonumber \\&\quad - \sum _\rho ^{3N_n} \frac{1}{M_\rho } \langle \Phi _I | \frac{\partial }{\partial R_\rho }|\Phi _J \rangle _{\textbf{r}}|_{R_\rho = \overline{R}_{\rho ,k,k'}^{(IJ)}} \langle \tilde{\chi }_{k }^{(I)} | \frac{\partial }{\partial R_\rho } | \tilde{\chi }_{k' }^{(J)} \rangle _{\textbf{R}} \, , \end{aligned}$$

where $\overline{\textbf{R}}_{k,k'}^{(IJ)}$ is the centroid position of the product of the two TBFs. The electronic-structure quantities—electronic energies and nonadiabatic coupling vectors —are evaluated only at $\overline{\textbf{R}}_{k,k'}^{(IJ)}$ for each pair of TBFs. We note that the second-order nonadiabatic couplings are neglected in the definition of the Hamiltonian matrix elements in AIMS (see Ref. [31] for additional information on this particular approximation).

The second approximation in AIMS is called *independent first-generation approximation* (IFGA) and proposes that the ancestor TBFs—the initial TBFs describing the nuclear wavefunction(s) at time t$_0$—can be considered uncoupled. This approximation is justified by the fact that a multidimensional wavepacket will rapidly spread following its creation by a short laser pulse, for example. This spreading would lead to a fast decoupling of the ancestor TBFs describing the initial nuclear wavefunction. It is important to note that the TBFs descending from a given ancestor TBF will all be mutually coupled, but the IFGA means that these TBFs are not coupled to the offspring of another ancestor TBF.

In summary, FMS proposes to represent nuclear wavefunctions using an adaptive basis of TBFs that evolve along classical trajectories and are coupled via Eq. (7). In the limit of a large number of TBFs, FMS would tend toward a numerically exact solution of the time-dependent Schrödinger equation. (We note that if the adiabatic representation is employed in FMS for the electronic states, additional challenges must be considered when describing the dynamics through a conical intersection—see Refs. [31–37].) AIMS uses the very same framework as FMS, but proposes to approximate the coupling between the TBFs (via the IFGA and SPA0), the quality and robustness of which have been tested extensively in the past [17, 19, 20, 25, 31, 38–42]. Thus, AIMS allows for ab initio nonadiabatic quantum molecular dynamics simulations of molecules in their full-dimensional space. We note that the current work will not discuss further the approximations in AIMS (the interested reader is referred to the references provided in this section) but will instead focus on the parameters emerging from the practical implementation of this method.

### Parameters in ab initio multiple spawning

The brief survey of the FMS and AIMS methods given in the previous section makes it clear that the use of AIMS, in particular, relies on the definition of a series of parameters. More specifically, we can define parameters that are directly linked to the AIMS method itself—the *method parameters* and others that are connected to its numerical implementation and/or efficiency considerations—*algorithm parameters*. In the following, we highlight the key parameters whose influence on AIMS dynamics will be tested in this work and connect them to their name in the code FMS90 [43].

The method parameters considered in this work will be the number of initial TBFs, the widths of the atoms forming the TBFs, and the nonadiabatic coupling threshold to initiate the spawning mode (CSThresh). For the algorithm parameters, we will study the influence of the time step (timestep), the minimum population of a TBF to be allowed to spawn a new (child) TBF (poptospawn), the overlap between a child TBF and the other TBFs during the spawning mode (omax), the screening of TBF overlaps to determine whether their Hamiltonian matrix element should be calculated (olapthresh), and the threshold for regularization when calculating the inverse of the overlap matrix in Eq. (6) (regthresh). Section 3 provides a detailed explanation of each of these parameters and their use in an AIMS dynamics.

### Molecular models used in this work: trans-azomethane and butratriene cation

Two dynamical processes were selected to study the impact that the parameters discussed in Sect. 2.2 may have on an AIMS dynamics: the photodynamics of trans-azomethane in full dimensionality and the nonadiabatic dynamics of the butatriene cation in a reduced-dimensionality model.

**Fig. 1:**
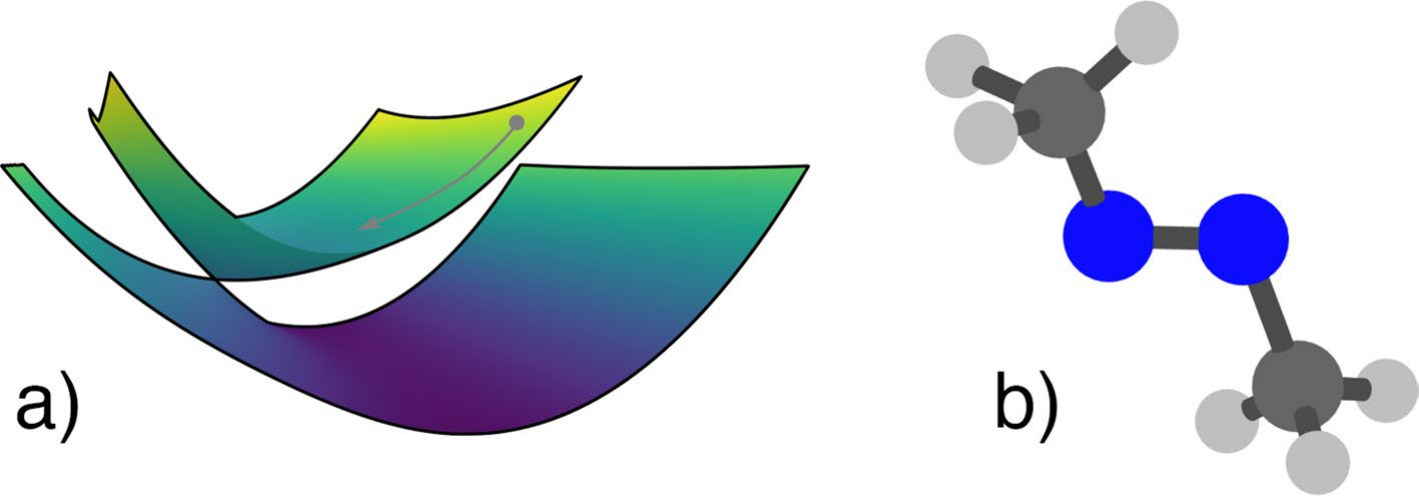
The two molecular models employed in this work to test the influence of parameters on an AIMS simulation. **a** Schematic depiction of the adiabatic potential energy surfaces for the butatriene cation. **b** Molecular structure of trans-azomethane

The photophysics and photochemistry of trans-azomethane, H$_{3}$C-N=N-CH$_3$ (Fig. 1b), has been studied with various methods for nonadiabatic dynamics. Persico pioneered our theoretical understanding of the photochemistry of azomethane by building model potential energy surfaces [44, 45], and using TSH simulations on their support to investigate the formation of photoproducts in gas phase and how such formation can be quenched in the presence of a solvent [46, 47]. Later, TSH simulations using on-the-fly electronic-structure calculations in gas phase and solution were conducted [48–51], as well as gas-phase AIMS dynamics [52, 53]. This series of works revealed that, in the gas phase, trans-azomethane photoexcited in S$_1$ ($n\pi ^*$) relaxes back to the ground electronic state S$_0$ in less than 500 fs. This decay is coupled with the formation of numerous photoproducts caused by isomerization and dissociation processes. The latter, taking place in S$_0$, first leads to the release of a first CH$_3$ from azomethane to form a methyldiazenyl radical, which rapidly dissociates into CH$_{3}$ + N$_{2}$. These processes appeared to be quenched in solution due to energy redistribution to the solvent, and only the isomerization route could be observed. Most recently, Persico and coworkers examined how the sampling technique for generating initial conditions influences the description of the aforementioned dissociation, and found that adding zero point energy to the PESs alleviated the zero point energy ’leakage’ problem affecting mixed-quantum classical dynamics [54]. In this work, we will focus on the fast nonadiabatic decay of trans-azomethane from S$_1$ to S$_0$ using AIMS coupled with SA-CASSCF to obtain on-the-fly the electronic-structure quantities required, as done in Ref. [52] (see Sect. 2.4 for the computational details).

We also used a two-dimensional two-state linear vibronic coupling (LVC) Hamiltonian model to investigate the photodynamics of the butatriene cation, H$_{2}$C=C=C=CH$_{2}^{+}$ (Fig. 1a) [36]. This two-dimensional model (see inset of following figures for a depiction of the two adiabatic potential energy surfaces) offers an opportunity to study the nonadiabatic dynamics of nuclear wavepacket in the vicinity of a sloped conical intersection. This model system also allows us to vary more broadly the range of each parameter studied.

### Computational details

#### Electronic structure

Electronic energies, nuclear gradients of the energies, and nonadiabatic couplings for trans-azomethane were calculated on the fly with state-averaged complete active space self-consistent field (SA-CASSCF) [55, 56] and a 6-31G$^*$ basis set [57, 58] within the MOLPRO 2012 program package [59–61]. A (6/4) active space was used—the two lone-pair orbitals on the nitrogen atoms, and the $\pi$ and $\pi ^*$ orbitals—with a state averaging procedure including the lowest two singlet states (S$_0$ and S$_1$). This level of electronic-structure theory was used in Ref. [52].

The parameters used for the two-state two-dimensional model system for the butatriene cation were obtained from Refs. [31, 36, 62].

#### Nuclear dynamics

For trans-azomethane, the same 40 initial conditions (ICs) are used for all AIMS dynamics and were sampled from a Wigner distribution of uncoupled harmonic oscillators, employing the normal modes of the minimum-energy ground-state geometry at the SA2-CASSCF(6/4)/6-31G$^*$. The reference AIMS simulation used the following parameters: standard width parameters [24]; time step (timestep) of 20 atomic time units (atu), further reduced to 5 atu in regions of nonadiabatic couplings; nonadiabatic coupling threshold to enter a spawning mode (csthresh) was fixed to 3.0 au$^{-1}$ (magnitude of the nonadiabatic coupling vectors); the minimum population of a TBF for it to be able to spawn a new TBF (poptospawn) was set to 0.01; the overlap between a child TBF and the other TBFs during the spawning mode (omax) was 0.6; screening of TBFs overlaps to calculate matrix elements (olapthresh) was 0.001; the threshold for regularization (regthresh) was set to 0.0001.

For the dynamics of the two-state two-dimensional model system for butatriene cation, the same 250 ICs are used for all AIMS dynamics and were obtained from a Wigner distribution of uncoupled harmonic oscillators centered at the Franck-Condon point in configuration space with a width (also used for the width of the TBFs) determined by the parameters of the model. [31] The reference AIMS simulation employed the following parameters: standard width parameters; time step (timestep) of 1 atu, further reduced to 0.25 atu in regions of nonadiabatic couplings; nonadiabatic coupling threshold to enter a spawning mode (csthresh) was fixed to 0.0001 au$^{-1}$ (magnitude of the nonadiabatic coupling vectors); the minimum population of a TBF for it to be allowed to spawn a new TBF (poptospawn) was set to 0.001; the overlap between a child TBF and the other TBFs during the spawning mode (omax) was 0.6; screening of TBFs overlaps to calculate matrix elements (olapthresh) was 0.001; the threshold for regularization (regthresh) was set to 0.0001.

What we take as the reference AIMS population trace (depicted by a black thick line in the upcoming plots) for the two systems under consideration in the following was obtained by using the set of parameters defined above. We stress that each population trace presented in this work results from a full AIMS simulation and not just the AIMS result obtained from a single initial condition.

The TSH [9] dynamics of trans-azomethane were performed with ABIN [63], interfaced with the MOLPRO2012 program package and using SA2-CASSCF(6/4)/6-31G$^*$. The same 40 ICs as for the AIMS dynamics were used, and each initial condition was repeated ten times (with a different seed for the random-number generator) to improve the convergence of the stochastic algorithm responsible for nonadiabatic transitions and the overall comparison with AIMS [22, 23, 64]. The time step was set to 20 atu. The energy decoherence correction (EDC) was employed with a default parameter of 0.1 au [64, 65]. The decoherence-corrected TSH is abbreviated dTSH in the following.

## Results and discussion

### Parameters related to the AIMS method

We first present our results for parameters that are directly related to the AIMS formalism itself, namely the number of initial conditions, the widths forming the TBFs, and the threshold to trigger the spawning mode.

**Fig. 2:**
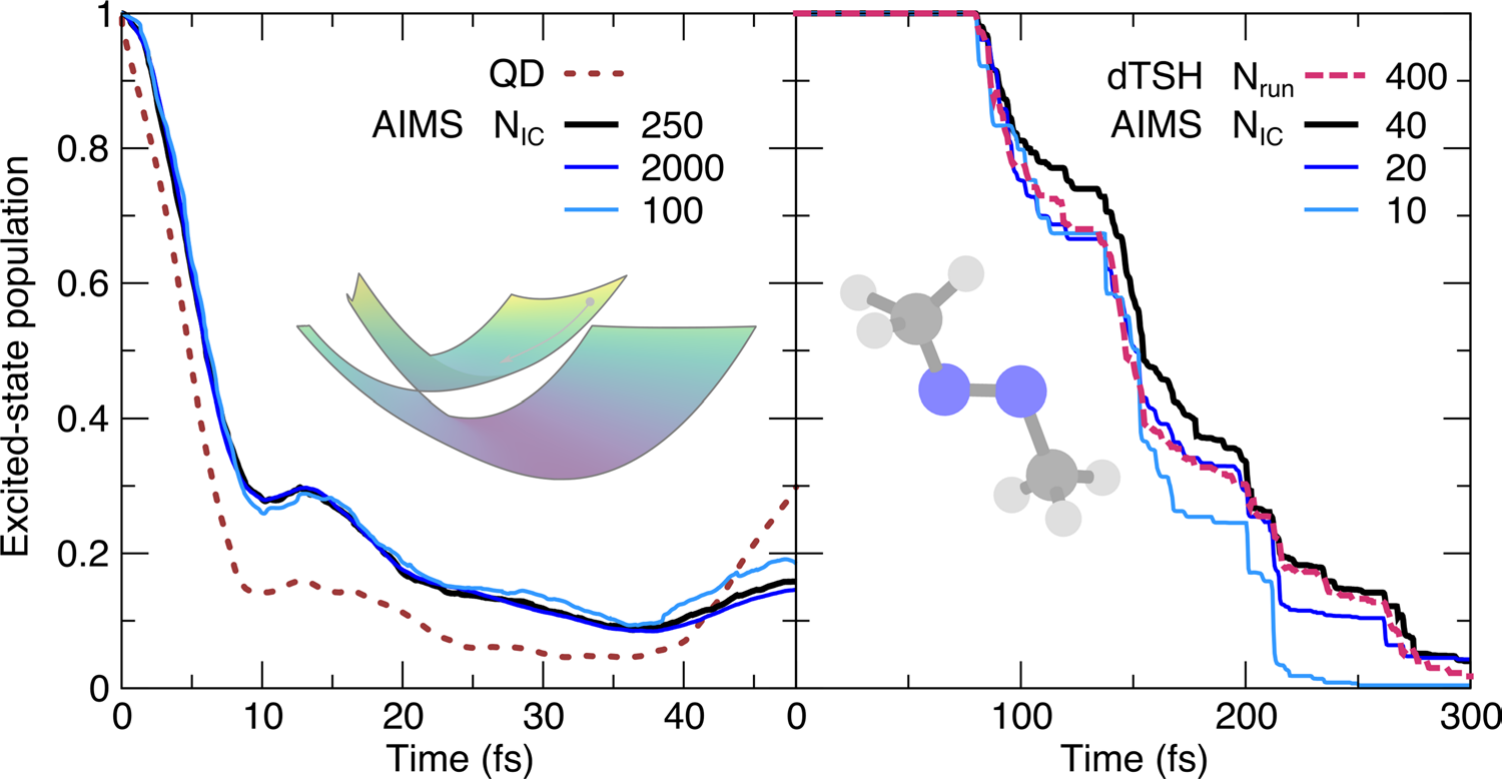
Influence of the number of initial conditions (N$_\text {IC}$) on the AIMS population trace (lines with different shades of blue), with the AIMS reference given by a black thick line. Left panel: population time traces for the butatriene cation model (inset). The result from a numerically-exact QD simulation is shown in dotted red. Right panel: population time traces for trans-azomethane (inset), with the population trace obtained from a dTSH dynamics shown in dashed red (400 runs, coming from 40 ICs each repeated 10 times)

The convergence of the population trace obtained in AIMS with respect to the *number of initial conditions* (N$_\text {IC}$) is presented in Fig. 2 for the butatriene cation model and trans-azomethane. The butatriene cation model allows us to sample a large number of ICs and indicates that the population trace obtained with 250 ICs is barely distinguishable from that with 2000 ICs (left panel of Fig. 2). Interestingly, even 100 ICs lead to a qualitatively converged result. The AIMS results overall qualitatively reproduce the quantum dynamics (QD) population trace for this challenging model of a slopped conical intersection, as observed in Refs. [31, 62]. For a molecular system in its full dimensionality (trans-azomethane, right panel of Fig. 2), a significant change in the population trace is observed from 10 to 20 ICs (in particular at time $t>125$fs). The smaller changes from 20 to 40 ICs indicate that convergence is being reached. We note that the population trace obtained in these simulations aligns with previously reported AIMS simulations employing 10 ICs [52]. Interestingly, TSH simulations using a decoherence correction and a swarm of 400 trajectories (the same 40 ICs as in AIMS, but each one was repeated 10 times—see Sect. 2.4.2) recovers the general trends of the AIMS population trace. The results obtained in this section are in line with earlier work comparing the convergence of AIMS with respect to the number of initial conditions [19, 22, 39, 42]. As each initial TBF will spawn new TBFs, the population trace obtained from an AIMS simulation may be expected to converge faster as a function of the number of initial conditions than that simulated with TSH [22].

**Fig. 3:**
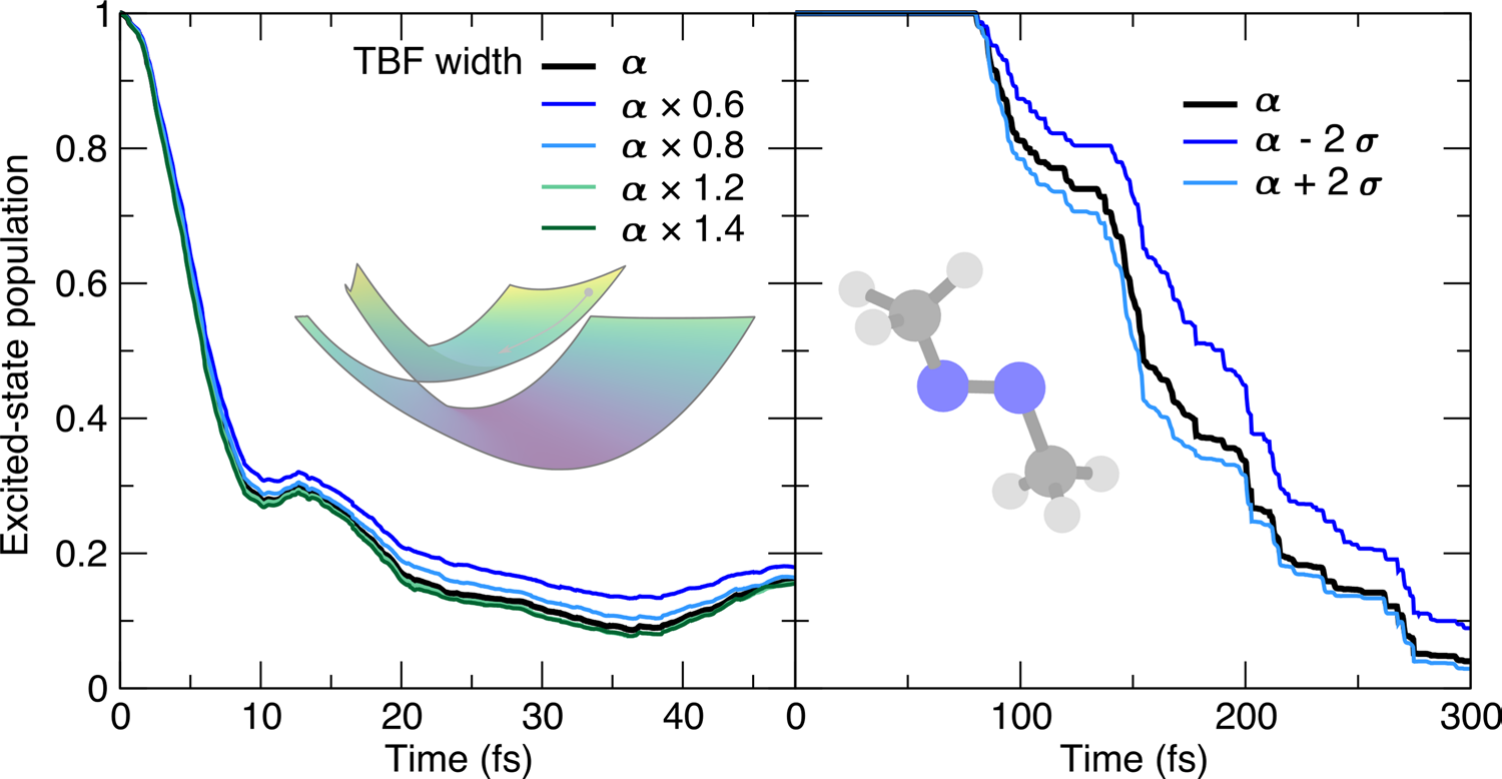
Influence of the width matrix for the TBFs ($\varvec{\alpha }$) on the AIMS population trace, with the AIMS reference given by a black thick line. Left panel: population time traces for the butatriene cation model (inset). Right panel: population time traces for trans-azomethane (inset)

We now turn to the sensitivity of an AIMS population trace as a function of the *widths of the TBFs*, $\varvec{\alpha }$ (Fig. 3). For the case of the butatriene cation model (left panel of Fig. 3), the widths for the TBFs were mapped to the ground-state vibrational wavefunction of the model. Multiplying these widths by a common factor only weakly influences the population trace for this two-dimensional model system, in line with earlier observations on model systems [25]. As discussed in Sect. 2.1, each atom in a full-dimensional molecule has a predefined width for its TBF component, and the widths of all atoms are grouped in the (diagonal) width matrix $\varvec{\alpha }$. The prescription to optimize the TBF widths is described in Ref. [24] and consists in maximizing the overlap between a reference ground-state vibrational wavefunction in internal coordinates and the FMS representation of a nuclear wavefunction (in Cartesian coordinates) for a series of molecules and determining an average width $\alpha _\nu$ for each atom $\nu$ with a corresponding standard deviation $\sigma _\nu$. Ref. [24] gives the following values for the atoms of interest here: $\alpha _\text {C}=22.7$, $\alpha _\text {N}=19.0$, $\alpha _\text {H}=4.7$ and $\sigma _\text {C}=6.3$, $\sigma _\text {N}=6.2$, $\sigma _\text {H}=0.7$ (all values in bohr$^{-2}$). To measure the variation in AIMS population trace, the width of each atom forming a TBF for trans-azomethane was then altered by $\pm 2\sigma _\nu$ (right panel of Fig. 3). Consistent with what was observed for the butatriene cation, the overall trend in the population decay is not substantially altered by this rather significant variation in width ($>50\%$ for the width of C and N).

The tests conducted here for both molecular systems also confirm an earlier observation that broader TBFs (smaller $\varvec{\alpha }$) appear to slow down the population transfer from the excited state, while slightly narrower TBFs (larger $\varvec{\alpha }$) speed it up [24]. As a side note, it is important to keep in mind that the use of broad TBFs may endanger the validity of the SPA0 approximation as it relies on a local overlap between coupled TBFs to justify a constant value for the electronic-structure quantity of interest in this region (for an example of the limits of the SPA0 in a similar context, the reader is referred to Ref. [20]).

**Fig. 4:**
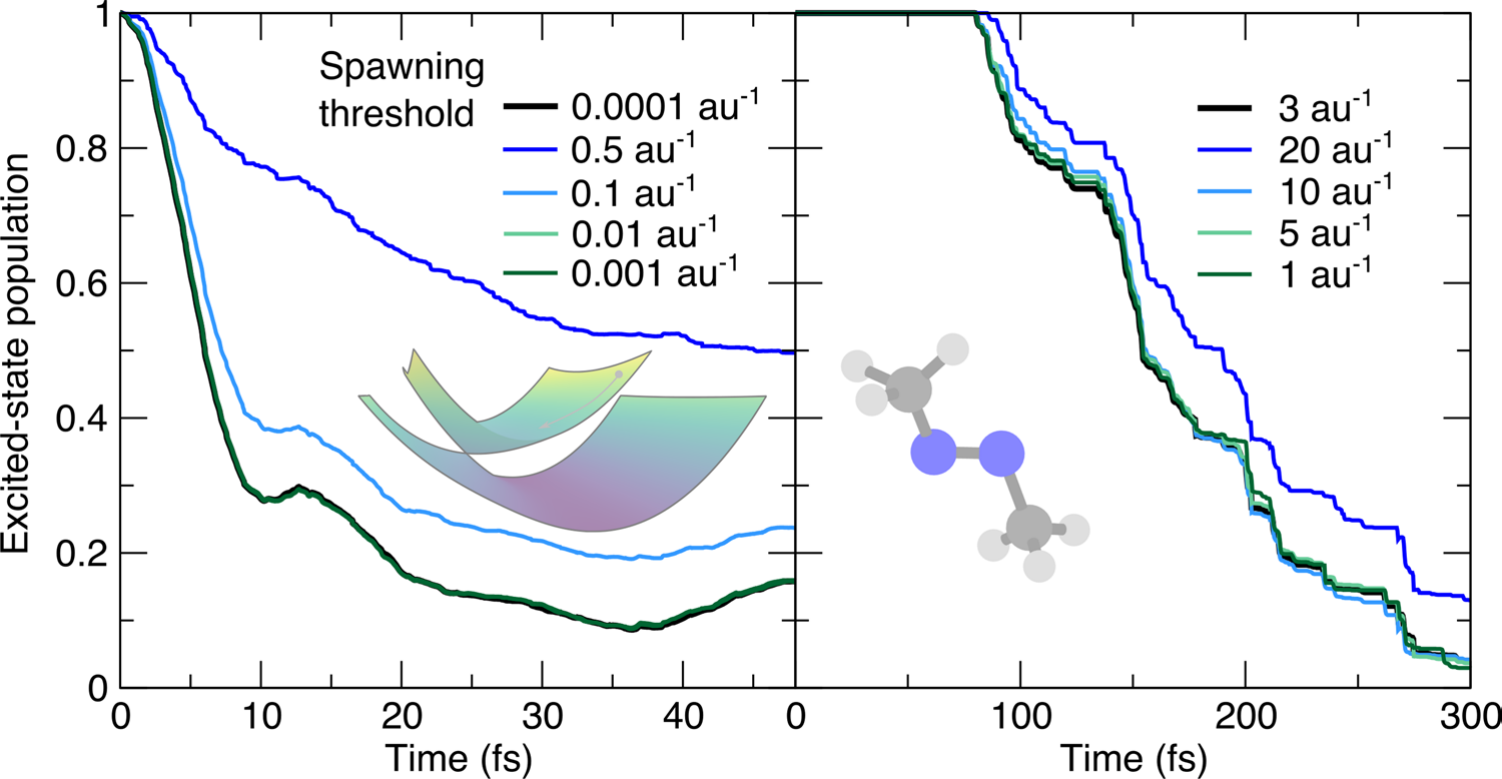
Influence of the nonadiabatic coupling threshold to initiate spawning (csthresh) on the AIMS population trace, with the AIMS reference given by a black thick line. Left panel: population time traces for the butatriene cation model (inset). Right panel: population time traces for trans-azomethane (inset). The coupling threshold is in both cases given by the magnitude of the nonadiabatic coupling vectors between the ground- and the first-excited electronic state

We conclude this section by focusing on the sensitivity of AIMS to the *nonadiabatic coupling threshold* to initiate the spawning mode (Fig. 4). This user-defined threshold is defined as the magnitude of nonadiabatic coupling vectors and compared to the nonadiabatic coupling vectors between the two electronic states of interests *I* and *J* at the position of the TBF *k* at time *t* evolving in electronic state *J*: $\left| \textbf{d}_{IJ}\left( \overline{\textbf{R}}_k^{(J)}(t)\right) \right|$. A very small value of the nonadiabatic coupling threshold means that the AIMS dynamics will often enter a spawning mode and possibly spawn new TBFs to capture any nonadiabatic events, even the weakest. While this behavior would in principle be ideal for the accuracy of the method, it also means that the number of TBFs will grow rapidly and that the majority of these TBFs may experience only a weak amplitude transfer thus contributing little to the overall transfer of population between electronic states.

A compromise is necessary to ensure that spawning occurs in the regions of configuration space visited by the TBFs that exhibit strong nonadiabatic coupling, but without spending too much computational effort, i.e., spawning TBFs in regions of weak nonadiabaticity. This behavior is clearly visible from the results of our simulations. In the case of the butatriene cation (left panel of Fig. 4), the population traces converge when a sufficiently small value of the nonadiabatic coupling threshold is employed. However, departing substantially from this required minimum value rapidly leads to a dramatic decrease in the rate of depopulation of the excited electronic state caused by the lack of new TBFs created (see blue and light blue curves in the left panel of Fig. 4). The AIMS dynamics of trans-azomethane follows a similar trend when varying the value of the nonadiabatic coupling threshold (right panel of Fig. 4). The reference value (black line, 3 au$^{-1}$) leads to a population trace nearly indistinguishable from that obtained with the smaller threshold (dark green line, 1 au$^{-1}$). A threshold of 20 au$^{-1}$ (blue line) is required to observe a deviation from the reference population trace. These results reinforce the findings of earlier works showing that the results of AIMS are stable with respect to the value of the nonadiabatic coupling threshold as long as this value is small enough to capture the important regions of nonadiabaticity [25, 28].

### Parameters related to the algorithmic implementation of AIMS

Our study moves on to the parameters in AIMS that are connected with the numerical implementation of the methods: time step, minimal population of a TBF for it to spawn, overlap of a newly-created child TBF with the swarm of existing TBFs, screening of the interaction between TBFs by their overlap, and the regularization threshold for the calculation of the inverse of the overlap matrix.

**Fig. 5:**
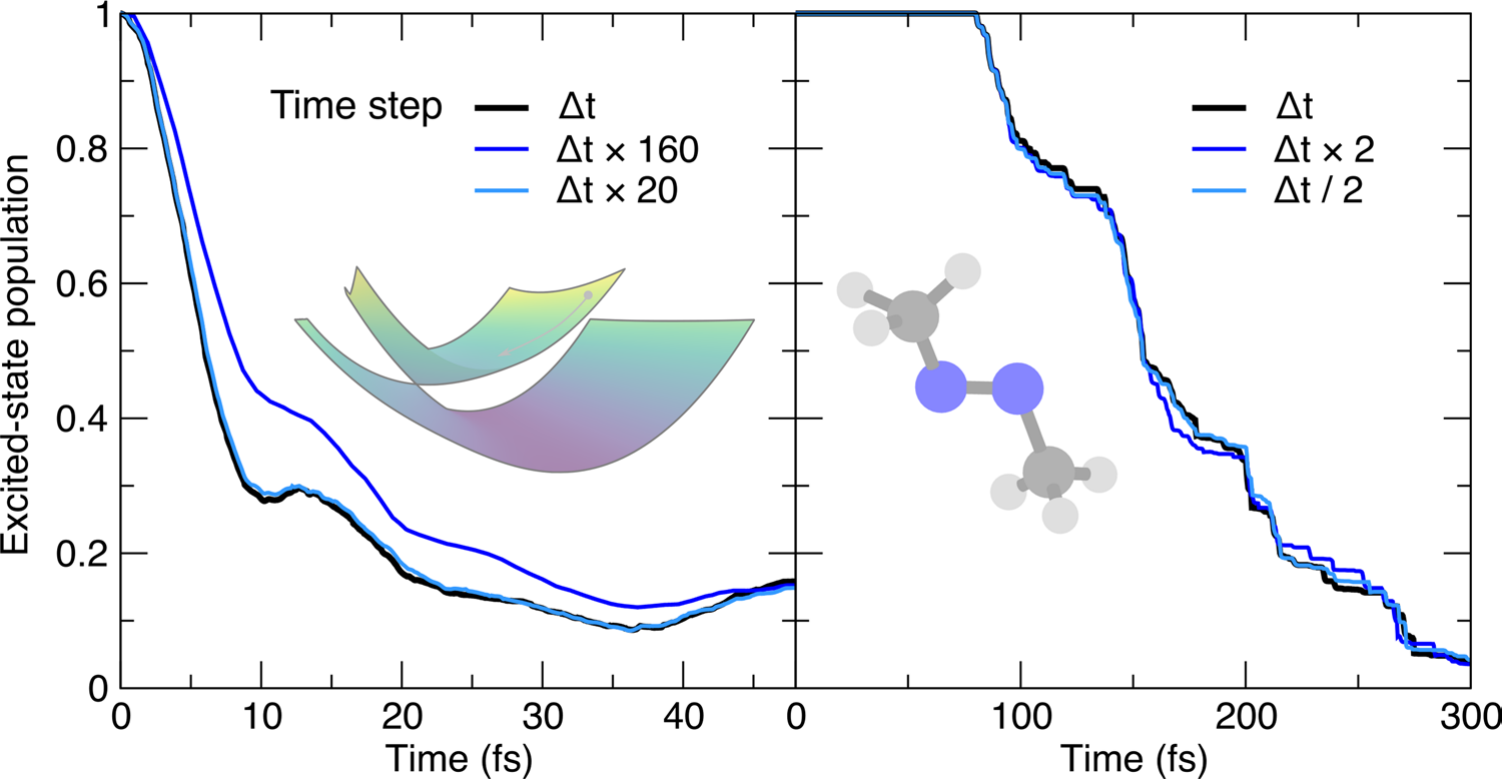
Influence of the nuclear time step (timestep) on the AIMS population trace, with the AIMS reference given by a black thick line. Left panel: population time traces for the butatriene cation model (inset). Right panel: population time traces for trans-azomethane (inset). The multiplicative factor indicated in the legend is applied to both the normal time step and the reduced time step (when TBFs are in a region of strong nonadiabaticity)

The integration of the classical equations of motion to propagate the TBFs are associated with a given (nuclear) time step. The current implementation of FMS90 uses an adaptive time step to ensure that the TBFs are propagated with an optimal time step in all circumstances (see Ref. [43] for the details of this implementation). A general time step is defined by the user and will be used for the adiabatic propagation of the TBFs, that is, when they are away from a region of strong nonadiabaticity. When TBFs reach a region with a sizable nonadiabatic coupling (for example, when a child and parent TBF are strongly coupled), the original time step is reduced (often by a factor of four) to ensure a proper integration of the underlying equations of motion for the complex coefficients (Eq. (6)). This reduction in time step is critical as the evaluation of the Hamiltonian matrix elements composing these equations of motion depends on the electronic-structure quantities determined along the classical propagation of the TBFs, and terms like the nonadiabatic coupling vectors may vary strongly with respect to nuclear coordinates in a nonadiabatic region. The switch to a reduced time step can also be triggered when a non-conservation of the norm or total energy over one integration step is detected, or when a TBF has possibly jumped over a nonadiabatic region. In this case, the integration step is rejected and the time step becomes adaptive, i.e., the time step is halved until the integration step is accepted (or the time step is smaller than a user-defined threshold, in which case the AIMS dynamics stops). The use of an adaptive time step leads to stable AIMS dynamics, as exemplified by the population traces for the butatriene cation and trans-azomethane (Fig. 5). Multiplying or dividing both the normal and the reduced time steps by a factor of two does not affect the population decay for trans-azomethane (Fig. 5, right panel). The model system of the butatriene cation allows us to visit more extreme values of the time steps and a multiplication by a factor $\times 160$ leads to a more substantial, yet not dramatic, variation of the excited-state population trace (Fig. 5, left panel).

**Fig. 6:**
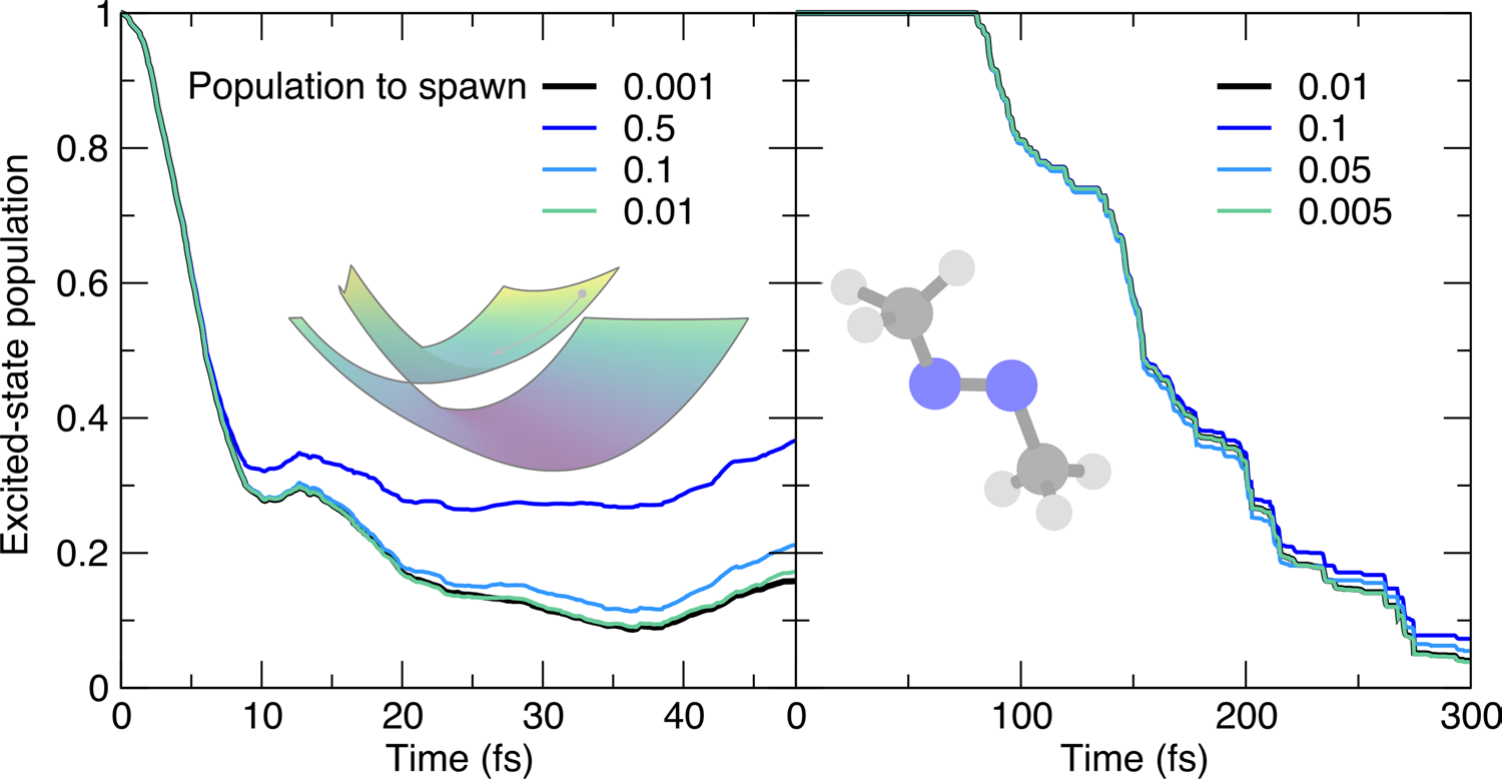
Influence of the minimal population that a TBF should be carrying to be allowed to spawn (poptospawn) on the AIMS population trace, with the AIMS reference given by a black thick line. Left panel: population time traces for the butatriene cation model (inset). Right panel: population time traces for trans-azomethane (inset). The parameter value corresponds to the minimal Mulliken population a TBF should carry to be allowed to spawn (see main text for definition)

Let us now focus on another parameter in AIMS: the minimal population that a TBF should be carrying to be allowed to spawn a new TBF. The cost of an AIMS calculation increases dramatically with the number of TBFs—the number of electronic-structure calculations required per time step is formally $N_{ES}=\frac{N_\textrm{TBFs}(t)\times (N_\textrm{TBFs}(t)+1)}{2}$ due to the calculation of the Hamiltonian matrix elements. Hence, different numerical strategies have been developed to minimize the unnecessary creation of new TBFs. A typical scenario one would like to avoid is to have a TBF *k* in electronic state *J* with a very small Mulliken population, defined in AIMS as $n_k^{J}(t)=\sum _{k'}^{N_\textrm{TBFs}^J(t)} \Re {\{C_{k'}^{(J)*}(t)S_{k'k}^{JJ}C_k^{(J)}(t)}\}$ [66], willing to spawn a new TBF on a coupled state. Such a spawning event is likely to not contribute to a significant amplitude transfer between electronic states but would still increase the computational cost of the AIMS simulation. A user-defined population can be set as a minimal threshold to permit a TBF to spawn new functions. This threshold has to be set with care as a too high value may result in nuclear amplitude being artificially trapped in an electronic state. This scenario is exemplified for the butatriene cation model, where small values of this parameter lead to similar excited-state population traces, while a value above 0.1 hampers the population transfer back to ground electronic state (Fig. 6, left panel). A similar trend is observed for the photodynamics of trans-azomethane, with close population traces for different small values of this parameter, but a trend for $t > 200$ fs toward a slow down of the population transfer correlated with the magnitude of the population threshold. We note that a stochastic-selection version of AIMS naturally alleviates the possible issues related to this parameter as unnecessary TBFs in the dynamics are automatically detected and stochastically discarded [62, 67–69].

**Fig. 7:**
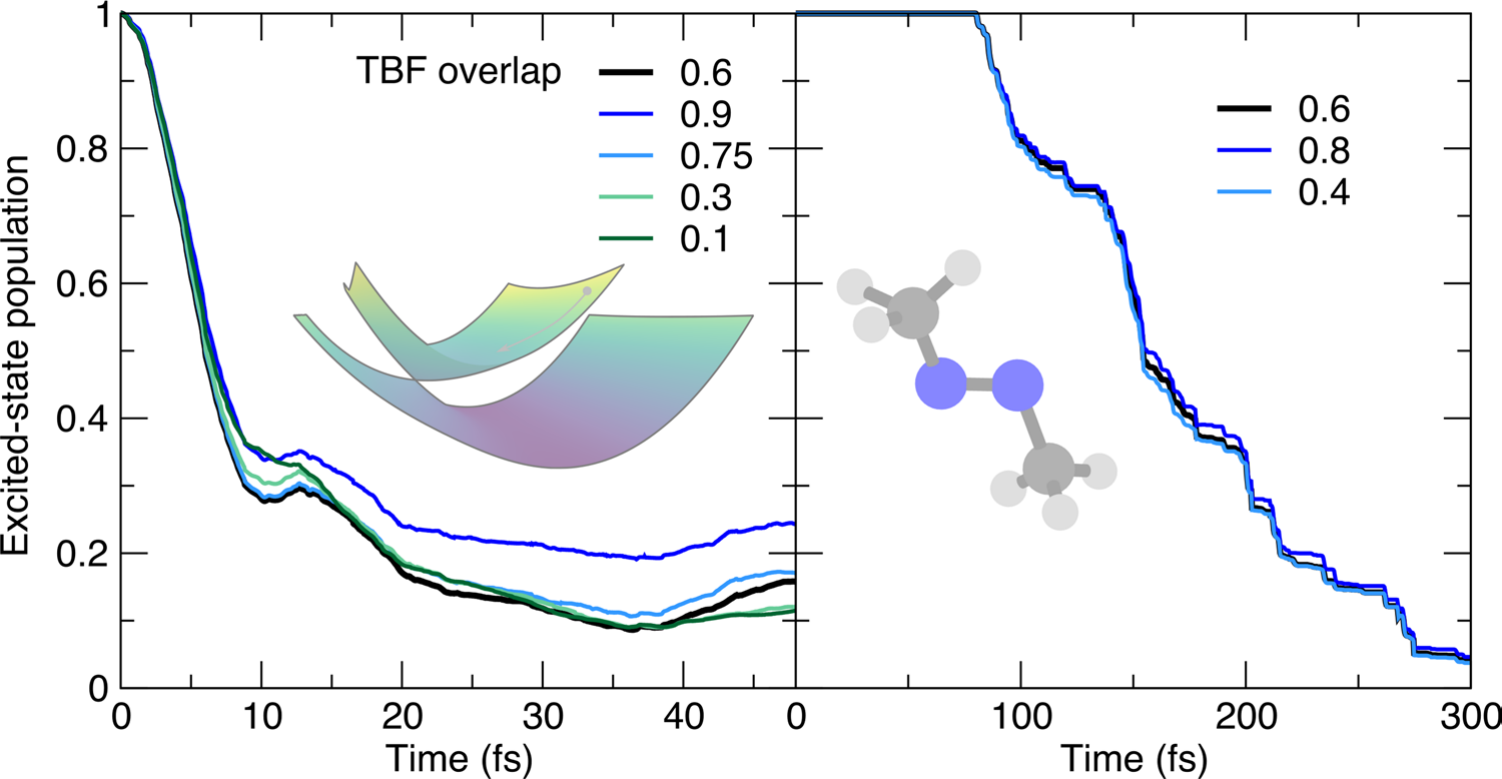
Influence of the threshold parameter for the overlap between a child TBF and its parent TBF (or the swarm) (omax) on the AIMS population trace, with the AIMS reference given by a black thick line. Left panel: population time traces for the butatriene cation model (inset). Right panel: population time traces for trans-azomethane (inset). The parameter value corresponds to the absolute value of the overlap between two TBFs

As stated in the previous paragraph, a series of parameters have been introduced in AIMS to ensure that any new TBFs spawned should benefit the overall nonadiabatic dynamics. An important factor to check then during the spawning process is whether (i) the parent and child TBFs have a large overlap at the spawning time^1^ and (ii) the child TBF exhibits a strong overlap with existing TBFs once backpropagated to the entry time of the spawning mode. In AIMS, a single user-defined threshold parameter is used to determine whether the overlap defined in cases (i) and (ii) is valid to justify the creation of the child TBF. The population decay for the trans-azomethane appears to be insensitive to the value of the overlap parameter around its reference value (Fig. 7, right panel). The model for the butatriene cation can be used to play more vigorously with the range of values for this overlap parameter and reveals that some deviations may happen for extreme values, caused by the dual nature of this parameter (Fig. 7, left panel). A large value for this parameter (e.g., omax $=0.9$ in Fig. 7, left panel) means that a child TBF is required to have a strong overlap with its parent for the spawning event to be accepted. Such a choice of value for the parameter may lead to spawning events being artificially rejected and consequently to a reduction of the population transfer (see blue curve in Fig. 7, left panel). This result indicates that the value of the parameter should be high enough to ensure a proper interaction between the child and parent TBFs, but not too high to prevent spawning events. This range for the selection of the overlap parameter also needs to consider the case (ii) described above: if the value of the overlap parameter is too low, that is, the child TBF is requested to only have a very minimal overlap with any other existing TBFs, the spawning process can also be aborted. This fact explains the trend of the population trace for omax $=0.1$ (dark green curve in Fig. 7, left panel), an overlap threshold too restrictive to permit the creation of new TBFs. Splitting the definition of this overlap parameter into two separate cases (overlap of the child TBF with its parent and overlap of the child TBF with the other TBFs) confirms the trends observed here (see Fig. S1 in the Supporting Information).

**Fig. 8:**
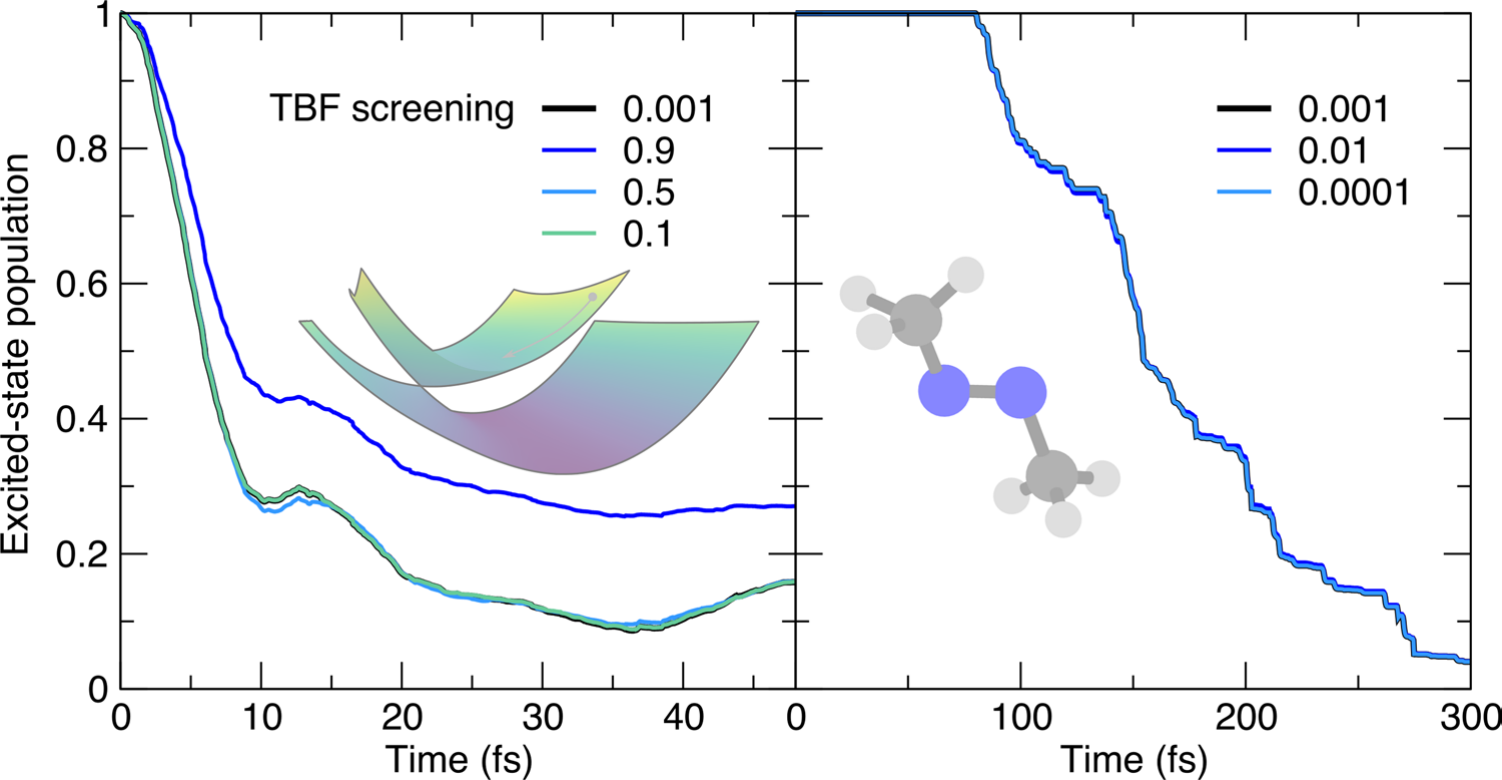
Influence of the threshold parameter determining whether matrix elements between TBFs should be calculated (olapthresh) on the AIMS population trace, with the AIMS reference given by a black thick line. Left panel: population time traces for the butatriene cation model (inset). Right panel: population time traces for trans-azomethane (inset). The parameter value corresponds to the absolute value of the overlap between two TBFs

The two previous paragraphs discussed numerical strategies to prevent uncontrolled and unnecessary growth of the number of TBFs. Investigating the Hamiltonian matrix elements used in AIMS (Eq. 8) reveals that the number of electronic-structure calculations per time step could be reduced by screening the overlap between TBFs and calculating matrix elements only for the TBF pairs with an overlap larger than a given threshold. This parameter is mostly hardcoded (threshold set to $10^{-3}$) and not usually changed by the user. The simulations conducted on both molecular models confirm the stability of the AIMS dynamics with respect to the choice of this threshold (Fig. 8). Only extreme values (0.9, blue curve in the left panel of Fig. 8) shows a noticeable deviation of the population decay from the reference by preventing an adequate coupling between TBFs. The stable results obtained for the butatriene cation with the large values 0.1 and 0.5 may appear surprising, but recent work on stochastic-selection AIMS highlighted the surprisingly low influence of TBFs couplings when their overlap value drop under 0.5 [68].

**Fig. 9:**
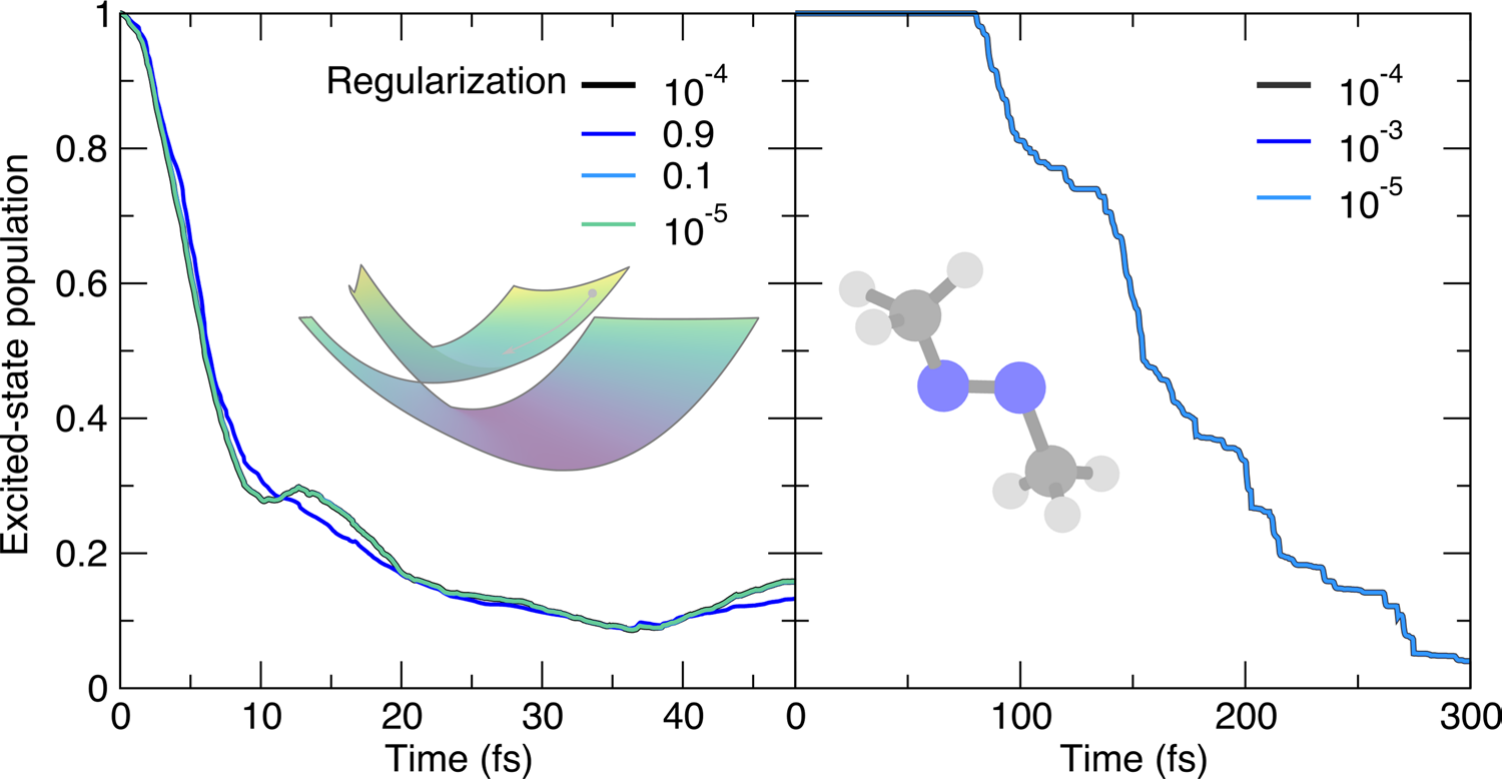
Influence of the threshold to regularize the overlap matrix before inversion (regthresh) on the AIMS population trace, with the AIMS reference given by a black thick line. Left panel: population time traces for the butatriene cation model (inset). Right panel: population time traces for trans-azomethane (inset). The parameter value corresponds to a threshold for the eigenvalue of the TBF overlap matrix

We conclude this section by monitoring how the regularization strategy employed for the inversion of the overlap matrix required by Eq. (6) can affect the result of an AIMS simulation. Substantial overlap between two TBFs (evolving on the same electronic state) can lead to linear dependencies in the overlap matrix. In turn, the inverse of the overlap matrix can become undetermined due to small (or zero) singular values of the overlap matrix, leading to issues with the propagation of the complex amplitudes with Eq. (6) [40, 70, 71]. In practice, the inversion is performed after regularization of the overlap matrix, where the threshold used to retain singular values is set to a predefined value ($10^{-4}$) [70]. All our AIMS simulations confirm the stability of the population dynamics as a function of the threshold employed (Fig. 9). Only a large value (0.9, blue curve on the left panel of Fig. 9) of the regularization threshold for the butatriene cation led to a deviation from the reference trace.

## Summary and conclusion

**Fig. 10:**
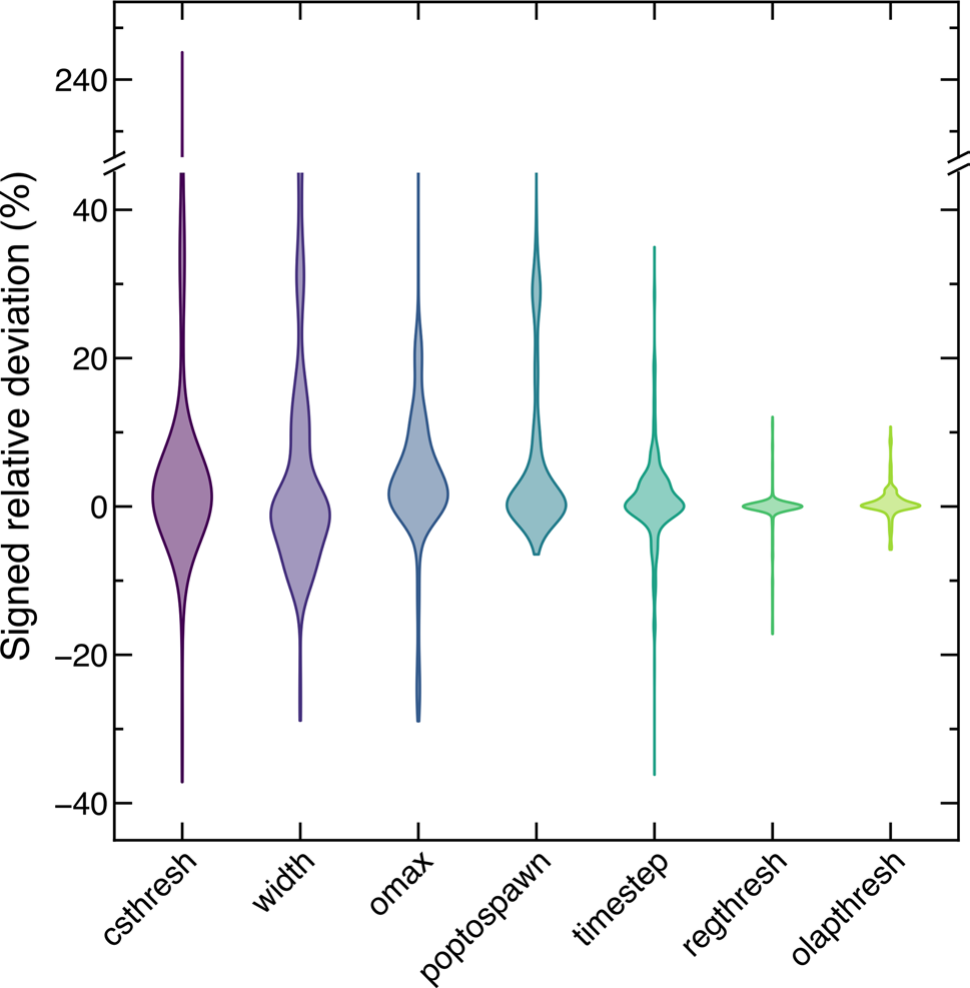
Kernel-density estimated probability distribution functions of the signed relative deviation in the excited-state population between the reference AIMS dynamics and the AIMS simulations employing different values for each individual parameter. The distributions were obtained by combining the AIMS results obtained for the two-dimensional butatriene cation model and the trans-azomethane

To conclude this work, we propose a visual summary of our results on the variability of the AIMS dynamics brought about by its parameters. To estimate the sensitivity of the AIMS method to a given parameter, we calculated the signed relative deviation of the excited-state population from the AIMS reference value at each time step and for each value of that parameter and both molecular systems studied (we note that the most extreme case for each parameter tested was excluded). The totality of the resulting data for each individual parameter was used to estimate the underlying probability density via a kernel density estimation using Scott’s factor for the bandwidth of the Gaussian kernel (see Fig. 10) [72]. Notice that the influence of the number of ICs was not analyzed in this way because this parameter has no effect at the level of a single AIMS run (for a given IC) but only on the average observables.

Focusing only on the fact that the signed relative deviation is mostly ±20 % for most parameters could let us conclude that AIMS is rather insensitive to the precise choice of its parameters. However, a careful analysis reveals that the total spread of the estimated probability distribution functions (EPDFs) increases drastically when looking at the parameters from right to left in Fig. 10—from $\sim 15$ % for the TBF screening threshold (olapthresh) to more than 200 % for the spawning threshold (csthresh). This observation naturally implies that choosing the right value for the latter is much more important than it is for the former. Furthermore, ordering the EPDFs of the parameters by their total spread reveals that the most important parameters are members of the *method* family: the spawning threshold (csthresh) and the TBF widths (width). The situation is not critical for the following reasons. First, the value of the TBF widths is set by a prescription —maximizing the overlap between a reference ground-state vibrational wavefunction expressed in internal coordinates and a nuclear wavefunction obtained from a product of complex Gaussians in Cartesian coordinates [24]. Hence, the values for TBF widths are optimized and, in line with earlier works, we do not recommend changing these values. When a reference width for a certain element is unavailable, a protocol can be used to determine it (see Refs. [24] and [73]). Second, we showed in Sect. 3.1 that the population trace obtained with AIMS converges for small values of the spawning parameter. To determine an optimal value for the spawning threshold, we advocate the following strategy: choose a small value for the threshold, perform a few AIMS runs starting from different ICs and plot the magnitude of the nonadiabatic couplings along the TBFs together with the chosen threshold. When the resulting plot shows that the nonadiabatic couplings are peaked and localized for a short amount of time, the spawning threshold should be taken to be small enough to ensure that new TBFs are included in the basis set at the beginning of each peak when the magnitude of the nonadiabatic coupling is still small. If, on the contrary, the magnitude of the nonadiabatic couplings monitored along the TBFs remains weak and spread out in time, there is often no way around choosing the spawning threshold only based on the computational resources available.

The two *algorithm* parameters that have the most influence on the population trace of our molecular systems are the overlap threshold between the child TBF and its parent TBF (or the swarm), omax, and the minimum population that a TBF needs to carry to be able to spawn, poptospawn. A good value for omax appears to be in the range of 0.4$-$0.6, as within that interval, the threshold seems to prevent redundant and suboptimal spawns without being too restrictive. When it comes to poptospawn, we recommend a threshold as small as possible, for example, 0.01 used as the AIMS reference value, to prevent a significant amount of the population from being trapped artificially in an electronic state that is not the ground state. Alternatively, this parameter could be set to zero by using a stochastic-selection variant of AIMS since there the TBFs with a low population will likely be removed from the simulation as soon as they become decoupled from the swarm. The AIMS result seems to be reasonably stable as a function of the other *algorithm* parameters—the time step, regularization threshold, and the TBF screening threshold. Based on our results, we could propose a more relaxed default for the TBF screening parameter olapthresh of 0.1 (instead of the current default set to $10^{-3}$), since the corresponding matrix elements appear to be negligibly small below that TBF overlap value and can be safely set to zero, saving some electronic-structure calculations. We stress that, in this work, we focused primarily on the excited-state population to gauge the variability of an AIMS simulation with respect to its parameters. This choice is motivated by the fact that most of the parameters are related to the process of transferring nuclear amplitude between TBFs. To gain more confidence in our conclusions, we performed a sensitivity analysis on the integrated absolute deviation in the (time-dependent) nuclear density for the two-state two-dimensional model system. We observed similar trends to what was observed for the electronic population (see Supplementary Information).

Importantly, the present work does not amount to a global sensitivity analysis [74, 75] of AIMS, which, to the authors’ best knowledge, is far from common in theoretical and computational chemistry. In this type of analysis, the input parameters are sampled in a representative way, and so-called sensitivity indices are calculated from the resulting output—these indices account for the correlation between the different parameters. In the present work, we only varied one parameter at a time while keeping the others fixed to the AIMS reference value, which means that correlations between the parameters are unaccounted for. Nevertheless, this independent analysis already yields valuable information on how each of the parameters affects the AIMS dynamics, as we have shown in this work, and it also constitutes the first step toward a global analysis. Note that such a thorough analysis would also necessitate sampling the parameters pertaining to the electronic-structure method—a challenge in itself.

In summary, this work offered an in-depth analysis of the different parameters used in the AIMS method for nonadiabatic molecular dynamics and to what extent changing their value affects the resulting AIMS dynamics. By simulating the photodynamics of two molecular systems—a two-level two-dimensional model of butatriene cation and trans-azomethane in its full dimensionality—and individually varying the parameters from their predefined baseline values, we found that the AIMS method is robust with respect to the choice of its parameters. An AIMS dynamics is, however, more sensitive to some parameters than others. We proposed clear signposts for choosing an optimal value for these parameters and hope that these will help users of the AIMS method for future simulations.

## Supplementary Information

Below is the link to the electronic supplementary material.

**Supplementary Information** The supplementary information contains a further analysis of the TBF overlap parameter \texttt{omax}, and an additional sensitivity analysis for the butatriene cation model. Initial conditions used for AIMS dynamics of trans-azomethane (ZIP). (pdf 964KB)

## Data Availability

Initial conditions used for AIMS dynamics of trans-azomethane are available in the SI (ZIP).

## Footnotes

1 It is important to note that the child TBF will be spawned at the exact same nuclear position as the parent TBF, but its nuclear momenta will be rescaled to account for total (classical) energy conservation, leading to a non-unitary overlap at the spawning point.
