# Supplementary material for: Probing the sensitivity of ab initio multiple spawning to its parameters
Source: Theor Chem Acc. 2023 Jul 28;142(8):66. doi: 10.1007/s00214-023-03004-w (PMC10382418; doi:10.1007/s00214-023-03004-w)
Supplement: Supplementary file 1 — Supplementary Information The supplementary information contains a further analysis of the TBF overlap parameter \texttt{omax}, and an additional sensitivity analysis for the butatriene cation model. Initial conditions used for AIMS dynamics of trans-azomethane (ZIP). (pdf 964KB) [file 214_2023_3004_MOESM1_ESM.pdf]

Supplementary information for:
Probing the sensitivity of ab initio multiple spawning to its parameters

Yorick Lassmann and Basile F. E. Curchod[a)]
Centre for Computational Chemistry, School of Chemistry, Cantock's Close, University of Bristol, Bristol BS8 1TS, UK

(Dated: 28 April 2023)

CONTENTS

I. Separation of TBF overlap threshold 2
II. Sensitivity analysis based on the nuclear density 3

[a)]Electronic mail: basile.curchod@bristol.ac.uk

I. SEPARATION OF TBF OVERLAP THRESHOLD

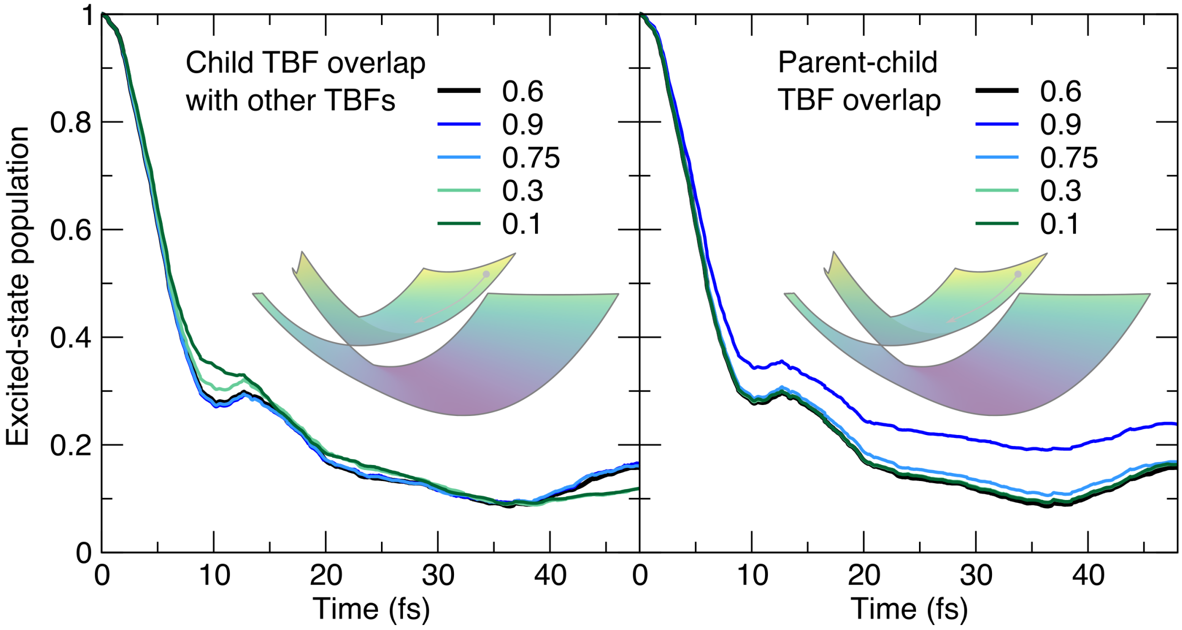

FIG. S1. Influence of the threshold parameter for the overlap between a child TBF and the rest of the swarm (left panel), and the parent TBF (right panel) on the AIMS population trace, with the AIMS reference given by a black thick line.

II. SENSITIVITY ANALYSIS BASED ON THE NUCLEAR DENSITY

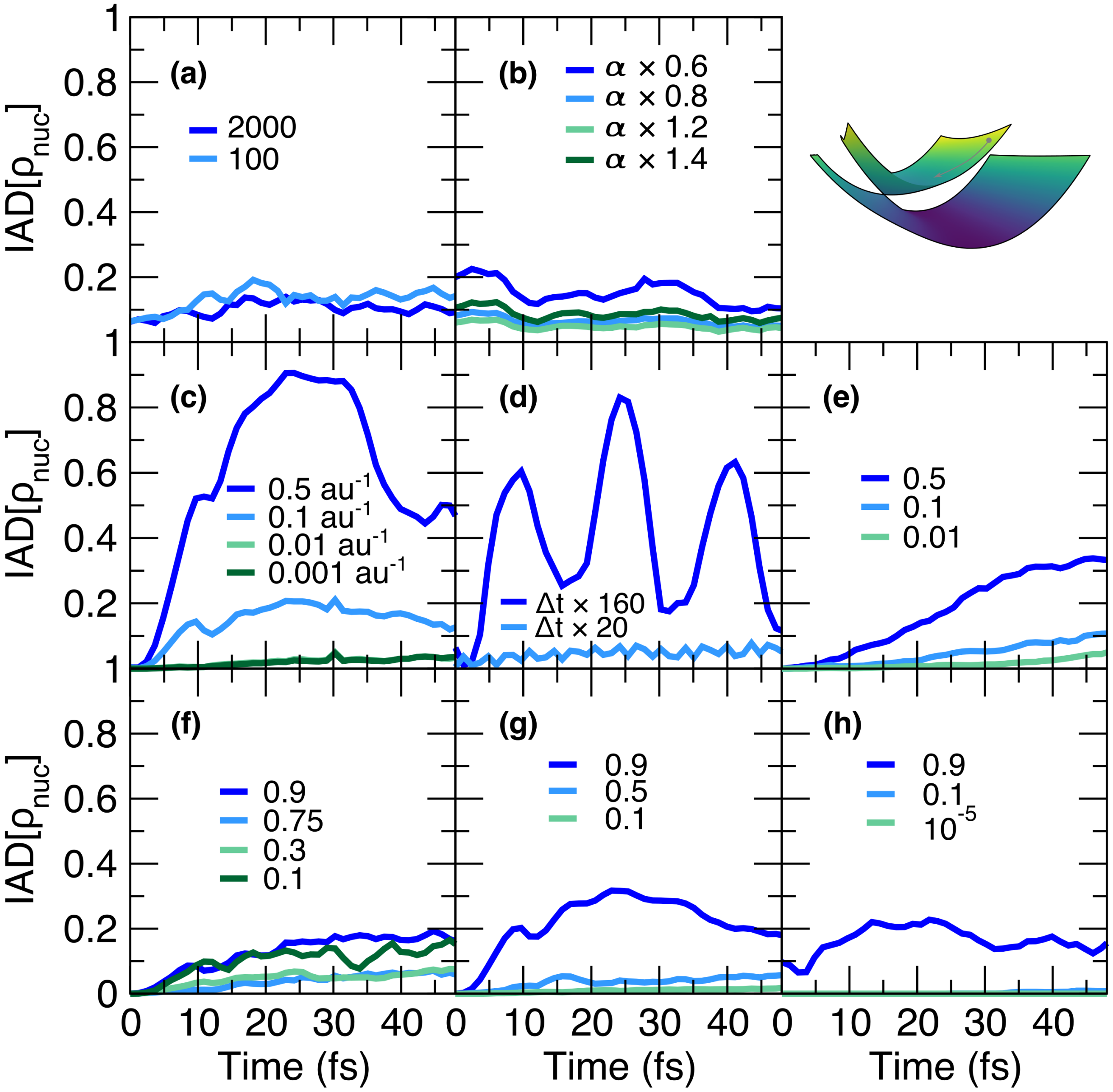

FIG. S2. Time traces of the integrated absolute deviation (IAD) in the (time-dependent) nuclear density, defined as $\mathsf{IAD}\left[\rho_{\mathsf{nuc}}\right](t) = \int_{-\infty}^{\infty} \mathsf{d}X \int_{-\infty}^{\infty} \mathsf{d}Y \left|\rho_{\mathsf{nuc}}(X,Y,t) - \rho_{\mathsf{nuc}}^{\mathsf{ref}}(X,Y,t)\right|$, with $\rho_{\mathsf{nuc}}^{\mathsf{ref}}(X,Y,t)$ being the AIMS reference nuclear density and $\rho_{\mathsf{nuc}}(X,Y,t)$ the nuclear density resulting from an AIMS simulation with the given parameter under study being altered. These simulations were conducted for the model system of the butatriene cation. The panels show the resulting IADs for the same parameters as in the main text, namely (a) number of initial conditions, (b) TBF width, (c) spawning threshold, (d) time step, (e) population to spawn, (f) TBF overlap, (g) TBF screening, and (h) regularization.
